# Microarray Based Diagnosis Profits from Better Documentation of Gene Expression Signatures

**DOI:** 10.1371/journal.pcbi.0040022

**Published:** 2008-02-15

**Authors:** Dennis Kostka, Rainer Spang

**Affiliations:** 1 Department of Computational Molecular Biology, Max Planck Institute for Molecular Genetics, Berlin, Germany; 2 Computational Diagnostics Group, Institute for Functional Genomics, University of Regensburg, Regensburg, Germany; University of Queensland, Australia

## Abstract

Microarray gene expression signatures hold great promise to improve diagnosis and prognosis of disease. However, current documentation standards of such signatures do not allow for an unambiguous application to study-external patients. This hinders independent evaluation, effectively delaying the use of signatures in clinical practice. Data from eight publicly available clinical microarray studies were analyzed and the consistency of study-internal with study-external diagnoses was evaluated. Study-external classifications were based on documented information only. Documenting a signature is conceptually different from reporting a list of genes. We show that even the exact quantitative specification of a classification rule alone does not define a signature unambiguously. We found that discrepancy between study-internal and study-external diagnoses can be as frequent as 30% (worst case) and 18% (median). By using the proposed documentation by value strategy, which documents quantitative preprocessing information, the median discrepancy was reduced to 1%. The process of evaluating microarray gene expression diagnostic signatures and bringing them to clinical practice can be substantially improved and made more reliable by better documentation of the signatures.

## Author Summary

It has been shown that microarray based gene expression signatures have the potential to be powerful tools for patient stratification, diagnosis of disease, prognosis of survival, assessment of risk group, and selection of treatment. However, documentation standards in current publications do not allow for a signature's unambiguous application to study-external patients. This hinders independent evaluation, effectively delaying the use of signatures in clinical practice. Based on eight clinical microarray studies, we show that common documentation standards have the following shortcoming: when using the documented information only, the same patient might receive a diagnosis different from the one he would have received in the original study. To address the problem, we derive a documentation protocol that reduces the ambiguity of diagnoses to a minimum. The resulting gain in consistency of study-internal versus study-external diagnosis is validated by statistical resampling analysis: using the proposed *documentation by value strategy*, the median inconsistency dropped from 18% to 1%. Software implementing the proposed method, as well as practical guidelines for using it, are provided. We conclude that the process of evaluating microarray gene expression diagnostic signatures and bringing them to clinical practice can be substantially improved and made more reliable by better documentation.

## Introduction

It has been shown that microarray based gene expression signatures have the potential to be powerful tools for patient stratification, diagnosis of disease, prognosis of survival, assessment of risk group and selection of treatment [1–3]. These signatures are computational rules for deriving a diagnosis from a patient's expression profile. Micorarray based molecular diagnosis constitutes a promising approach, but so far it has been difficult to exploit its potential. Before gene expression signatures can impact clinical practice, they need to be communicated to other health care centers with data for external evaluation [2], and ultimately to practitioners for use in clinical routine. This requires unambiguous documentation of the signature.

We believe documentation does not receive sufficient attention. For instance, it has been observed that the reconstruction of published signatures can require an expert re-analysis of the study [4] or may not be possible at all [5]. Documenting a signature is conceptually different from reporting a list of genes contributing to the classification rule, since the latter does not determine how an external patient should be diagnosed. Moreover, we show that even the exact quantitative specification of the classification rule does not constitute a ready-to-use signature. In addition, a procedure transforming raw external expression data to the signature-specific scale has to be provided.

Based on eight clinical microarray studies [6–13] we show that common documentation standards have the following shortcoming: When using the documented information only, a study-external patient might receive a diagnosis different from the one he would have received as part of the original study. To address the problem, we propose guidelines and methodology for building and documenting signatures unambiguously. We demonstrate that our approach reduces ambiguity of diagnoses to a minimum.

## Results

### Ambiguity of Diagnosis

Before we start with a systematic analysis of the signature documentation problem, we illustrate it in the context of the breast cancer study of Huang et al. [9]. This study includes expression profiles from 89 breast cancer patients. The majority (34) had no recurrence of disease, 18 suffered from a relapse and for the rest no data about recurrence is available. We preprocessed the entire study data using the rma protocol (see Methods), and derived a prognostic signature using a subset of the data (training set). Patient 00291471 was not part of the training set. When applying the signature, this patient was predicted to be relapse free. Next, we classified patient 00291471 a second time, this time as a study-external patient from another health care center. Consequently, the expression profile was preprocessed independently from the study data but using the same preprocessing protocol (rma). Again, we applied the prognostic signature, and this time the patient was predicted to relapse. The reason for this inconsistency is that any quantitative specification of the classification rule critically depends on the scale of the preprocessed data. The scale, in turn, depends on the data included in the preprocessing process, which is different in an internal and an external analysis.

### Documentation Strategies

We studied the signature documentation problem more systematically on eight clinical microarray studies [6–13] involving different cancer types and representing diagnostic as well as prognostic classification problems. Table 1 gives an overview. We compared two different documentation strategies:

**Table 1:**
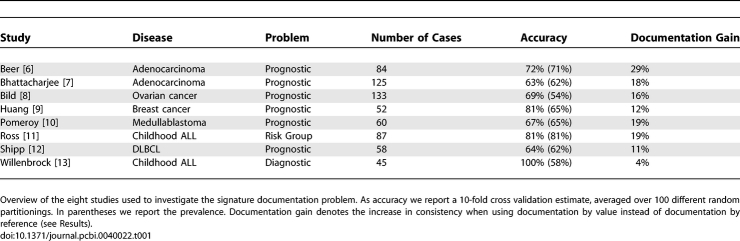
Summary of Microarray Studies

#### Documentation by reference.

The signature is documented by a quantitative classification rule and by referencing the underlying preprocessing protocol. This includes the bookkeeping of user-adjustable parameters, such as the target scale for the standard Affymetrix protocol (mas). External cases are preprocessed by running the specified procedure exclusively on the new data.

#### Documentation by value.

The signature is documented by a quantitative classification rule and by supplying a set of parameters (values) determined by the fitted vsn and rma preprocessing procedures (see Methods). These parameters depend on the data that was preprocessed and determine its scale. External cases are scaled by using the corresponding add-on preprocessing routines (see Methods).

Documentation by reference does not include properties of the study data. A comparable scale is assumed to be generated automatically, given the exact details of the preprocessing routine. In contrast, documentation by value communicates the scale implied by the study data explicitly. Data normalized by rma was documented using both strategies. In case of vsn, normalized data was documented by value only. The reason is that documentation by reference requires applying the preprocessing protocol to a single array. In the case of vsn, the normalization step requires more than one array, so this is not possible. On the other hand, documentation by value works well in this setting. Finally, for the Affymetrix Microarray Analysis Suite the two strategies coincide, since arrays are normalized independently of each other.

### Consistency of Diagnosis

We calculated consistency and kappa index as described in the Methods Section. To that end, we repeatedly split each dataset into an internal set (of size 20, 30 and 40) to derive the signature and an external set for validation (using documented information only). This mimics the process of communicating signatures between health care centers. The validation results were compared to a reference diagnosis and the fraction of agreement is reported as consistency. This procedure requires the encoding (documentation) of signature information and its subsequent decoding for use on external data.

The main results are summarized in Table 2, the complete details can be found in supplemental Figures S1 to S8. First, we observed in the study by Beer et al. [6] that documentation by reference can lead to discrepancies between external and internal diagnosis being as frequent as 30%. The median consistency across all studies using documentation by reference was 82%, corresponding to a median discrepancy of diagnoses as high as 18%. This demonstrates the existence and importance of the documentation problem. External researchers will generally not obtain the same diagnoses as the investigators of the original study. More importantly, we observed that the documentation strategy matters: Documenting signatures by value leads to substantially more consistent results than documentation by reference. We observed the biggest effect for the prognostic study by Beer et al., where consistency improved from 70% to 99%. This corresponds to a consistency gain between 27% and 31% (95% CI).

**Table 2:**
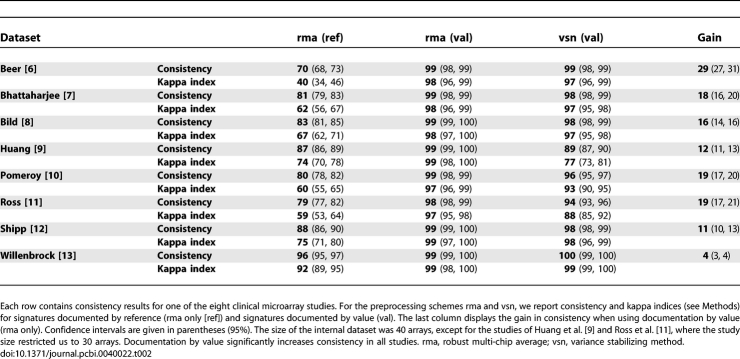
Documentation by Value Increases Consistency

The smallest consistency gain (between 3% and 4%, 95% CI) was observed for the diagnostic study of Willenbrock et al. [13], which poses the easiest classification problem. The median minimal gain in consistency (97.5 % CI) obtained from documenting signatures by value was 15%. On most datasets consistency of rma and vsn preprocessing were comparable; the differences were small. Exceptions are the datasets of Huang et al. [9] and Ross et al. [11], where consistencies obtained with rma were larger. Overall, signatures documented by value displayed high consistency, most of them larger than 98%. Documentation by reference was found to be significantly less consistent (about 15% median consistency loss). Surprisingly, we see no big effect of the size of the internal set on the observed consistency (Supplemental Figures S1-S8). Also, the two learning algorithms (SVM and shrunken centroids, see Methods) seem to perform comparable with respect to consistency (Figures S1-S8). Note that for data normalized with the Affymetrix Microarray Analysis Suite the consistency problem does not exist. Since arrays are normalized independently of each other, one is guaranteed to receive 100% consistency.

## Discussion

To the best of our knowledge, this is the first study investigating the problem of documenting diagnostic expression signatures. We were able to demonstrate that common documentation standards are insufficient for unambiguously determining diagnosis. Moreover, we have shown that the consistency of diagnostic signatures can be substantially improved by documenting data-dependent preprocessing information. To do so, we have proposed the documentation by value strategy.

We observed a trade-off between the reported performance of preprocessing protocols and the effort required for documenting them [14,15]. While it is known that preprocessing schemes that share information across arrays enhance precision and accuracy of estimated expression differences, improved normalization performance comes at a price: The already normalized expression values for a fixed microarray change when additional arrays are added to the study. This is a problem for applying a signature to external data; the original data needs to be included in the normalization of external arrays. The re-normalization of the complete dataset changes the original expression values, affecting the signature and the molecular diagnosis of patients in the original study. To circumvent this problem, we have altered the widely used preprocessing methods rma [16] and vsn [17] to provide an add-on mode. This allows one to first process a core dataset, and then to add data from additional arrays without changing the normalized core data.

As a summary of our findings we propose the following guidelines for deriving and documenting a diagnostic gene expression signature:

### Preprocessing

Preprocess the data using a protocol that allows for later inclusion of arrays without changing the original expression values. For example, this can be done preprocessing arrays independently of each other, or by making use of the software we provide.

### Building the Classification Rule

Derive a classification rule using software that provides a complete quantitative specification of the signature for documentation purposes. For example, this can be the nearest shrunken centroid procedure [18] we employed.

### Documentation by Value

Document the full quantitative specification of the classification rule. In addition, document preprocessing by including aggregate data-dependent information. For example, this can be done with the software we provide. Ideally, make both parts available as an integrated open source computer program that can readily be used to diagnose new patients.

### Diagnosing an External Patient

Bring the raw data to a signature consistent scale. For example by using our software. Apply the documented classification rule to diagnose the new patient.

These guidelines suggest methods we have found to work well in practice, but we do not claim them to be optimal in any sense. Given the heterogeneity of clinical data as well as the diversity of array platforms, it can safely be assumed that there is data where other methods are more appropriate. However, we believe that in these situations the documentation problem still exists, and a similar documentation by value strategy should be developed for the methodology in use.

In our simulation setup, the data we call external are actually arrays from the same study. With real external data, additional problems occur. It has been shown that even when using the same technology and experimental protocols, the resulting data for the same tissue sample varies between different health care centers [15,19]. Also, different centers might not employ the same definitions of outcomes and/or diagnoses. While this effect is not directly linked to documentation, we believe that the benefits of documenting signatures by value are enhanced in situations where external and internal data are more heterogeneous.

Documentation of signatures is significantly easier for preprocessing methods treating arrays independently of each other, as is the case for the Affymetrix Microarray Suite. However, preprocessing protocols sharing information across different arrays are commonly used as they can provide increased accuracy [15]. Overall, methods like mas present a viable alternative to more advanced procedures whenever consistency is of prime importance, as it is the case in clinical microarray studies. In our study we did not find strong evidence favoring one approach over the other.

While microarray based diagnostic signatures hold great promise to improve diagnosis and prognosis of disease, evaluation of a signature's predictive performance is difficult and subject to current research and argument [4,19–23]. It is important to prove that a signature holds independent complementary information to existing prognostic markers. While sharing candidate signatures within the research community can accelerate the process of evaluation, this does not allow for any ambiguity of signatures. We believe that our documentation by value strategy removes this obstacle and greatly facilitates signature evaluation. The additional effort required for documentation by value is small. There are certainly several ways to implement documentation by value, and we provide suggestions for two preprocessing schemes. Overall, we found that the consistency of diagnoses based on gene expression diagnostic signatures critically depends on the documentation of the signature. Optimal consistency was obtained by using the proposed documentation by value strategy.

## Methods

### Data sources.

We studied the signature documentation problem on eight clinical microarray studies [6–13], involving different disease types and representing diagnostic as well as prognostic classification problems. See Table 1 for an overview.

### Preprocessing.

Raw microarray data is subject to noise. There is variation in the data that is not due to biological signal, but rather to measurement error or experimental artifacts. Prior to data analysis it is therefore common practice to perform data preprocessing. Preprocessing procedures depend on the microarray technology in use. We focused on Affymetrix GeneChip technology, where data preprocessing commonly includes three steps: Background correction, normalization and probeset summary. We focus on three of the most widely used protocols: Standard preprocessing as provided by the Affymetrix Microarray Analysis Suite Version 5 (mas) [24], a procedure called ”robust multi-chip average” (rma) [16], and a variance stabilizing method (vsn) [17]. For vsn and rma we provide an ”add-on” mode. This mode enables one to first process a core dataset, and then to add data from additional arrays without changing the normalized core data.

### Learning algorithms.

In order to achieve comparable results across studies, we re-learned classification rules using the same two learning algorithms for all datasets. We chose a nearest shrunken centroid classifier (pam) [18] that was shown to perform well in recent comparisons of classification algorithms [20,25]. Additionally we used support vector machines (SVMs) [26] with a linear kernel. In the case of pam, classification rules were documented by gene-specific weights and class-specific shrunken centroids. For the SVMs gene specific scaling factors, the support vectors and the offset were documented. All these parameters depend on the scale of the preprocessed data.

### Consistency.

Consistency of a signature was assessed through re-learning, using 1000 different random subsets of the study data (sub-sampling), and then analyzing the diagnoses of patients across all sub-sampling runs. We randomly split each of the eight datasets into two parts, which we call the internal and the external set. The size of the internal sets was fixed to 20, 30 and 40 arrays for all datasets. For a given preprocessing and documentation strategy (see Results), we first derived and documented a signature using the internal set. Then we applied this signature to a random sample of the external set, exclusively using the information documented beforehand. This mimics the process of communicating signatures between health care centers.

For evaluation of performance we determined the diagnosis a patient would have received if analyzed in the context of the original study (reference diagnosis). To this end, we concatenated each external case with the (say *N* ) internal arrays, renormalized this complete dataset of *N + 1* cases and applied the signature to the external case. The result constitutes the reference diagnosis for this patient. Then we compared all prior diagnoses from the sub-sampling runs to the reference. The fraction of matching diagnoses was reported as a consistency index. A consistency of one corresponds to the situation where all diagnoses were identical to the reference. A consistency of zero implies that all diagnoses were different from the reference. In addition we report the kappa index [27], a statistical measure to assess inter-rater reliability.

### Statistical analysis.

All confidence intervals were calculated assuming Bernoulli models for class predictions. In the case of confidence intervals for consistency gains, an additional convolution of estimated Binomial densities was carried out. More details can be found in supplemental material.

### Signature documentation.

In the core of this paper lies the documentation by value strategy for documenting diagnostic signatures: In addition to the parameters describing a classification rule, documentation by value also keeps track of the normalization dependent scale that is underlying the signature. This scale does not only depend on the preprocessing strategy, but also on the original data. In the following, we demonstrate how to document the scale for two preprocessing methods, rma and vsn.

### Documenting quantile normalization.

For rma, background correction is performed on an array-by-array basis. The subsequent normalization step can be documented as follows. Assume we have *p* probes and *n* arrays. Let *X* be the *p* × *n* background corrected probe-level expression matrix on log scale. Let *π* be the permutation sorting the columns of *X* and *π* ^−1^ its inverse. Then the quantile normalized [28] version of 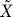 of *X* is defined as:

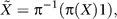
where 1 is a *n* × *p* matrix with all elements equal to 1/*n*. Let 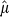 be equal to the first (and therefore any) column of *π*(*X*)1. Then 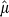 is the vector of (identical) quantiles of each normalized array. To bring an external array to this scale, let *x* ∈ ℜ^*p*^ be its raw probe level expression values. If *π_x_* is the permutation sorting the entries of *x*, a scale-consistent version of *x* is given by

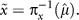



The normalized array 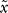 is consistent with the scale of the other arrays as it has the same quantiles. Since *π_x_* depends on the sorting of the entries of *x* only, we do not need to worry about a global background correction. That is, up to probeset summary, rma can be documented by keeping track of 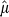 .

### Documenting the variance stabilizing transformation.

In the case of vsn, the raw probe-level expression matrix *X* is background corrected and normalized simultaneously. Huber et al. [17] relate random variables *X_ki_* (*k* = 1 . . . *p* and *i* = 1 . . . *n*) to the true abundance *μ_k_* of probe *k* on array *i*, given probe *k* is not differentially expressed:

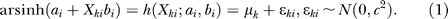



The parameters *a_i_* ∈ ℜℜ, *b_i_* ∈ 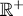 and *c* ∈ 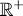 are estimated from the data.

Assume vsn normalized core data is at hand. For *n* arrays we have normalized expression values 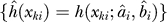 with *i* = 1 . . . *n* and *k* = 1 . . .*p*, and corresponding parameter estimates 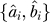 . Also, a set *K* of not differentially expressed genes has been identified. This implies estimates 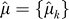 for each gene *k* ɛ *K* and an estimate of the variance of the residuals in (1):

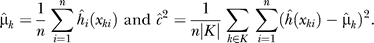



We want to transform an external sample *x^e^* to the scale determined by the *n* core arrays. By employing the same model as for the core data (Equation (1)) and plugging in the estimates, we get maximum likelihood estimators for the parameters *a^e^* and *b^e^*:

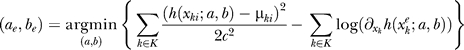
which can be calculated numerically. These estimators are completely determined by *μ̂*,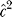 and measurement values from the external array. They define the variance stabilizing transformation 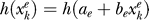 bringing *x^e^* to the same scale as the core data. Therefore, up to probeset summary, vsn preprocessing is fully documented by *μ̂* and 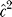 .

### Documenting an additive model for probeset summary.

Let *X^(k)^* be the submatrix of normalized expression values indexed by the probes belonging to probe set *k* (across all arrays). Let there be *l* probes in probeset *k*. Then an additive model assumes

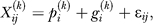
where 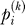 , *i* = 1 . . . *l*, is a probe specific effect and 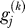 , *j* = 1 . . . *n*, represents the abundance of mRNA of gene *k* on array *j*. The parameters 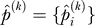 and 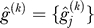 can e.g. be estimated by the median polish procedure [29]. Denote by *x^e^* a (suitably normalized) external array. Let *x* ^*e,*(*k*)^ denote the values for probeset *k*. Then median (*x^e,(k)^* − 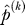 ) denotes a consistent estimate of the expression of gene *k*. That is, the additive model is fully documented keeping track of the probe effects 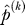 for all probesets on the array.

## Supporting Information

Figure S1 Beer et al. Beer et al. [6].
(16 KB PDF)

Figure S2 Bhattacharjee et al. Bhattacharjee et al. [7].
(16 KB PDF)

Figure S3 Bild et al. Bild et al. [8].
(16 KB PDF)

Figure S4 Huang et al. Huang et al. [9].
(15 KB PDF)

Figure S5 Pomeroy et al. Pomeroy et al. [10].
(16 KB PDF)

Figure S6 Ross et al. Ross et al. [11].
(15 KB PDF)

Figure S7 Shipp et al. Shipp et al. [12].
(16 KB PDF)

Figure S8 Willenbrock et al. Willenbrock et al. [13].
(16 KB PDF)

Text S1 Supplementary Methods and Software (225 KB PDF)
