# Supplementary material for: Microarray Based Diagnosis Profits from Better Documentation of Gene Expression Signatures
Source: PLoS Comput Biol. 2008 Feb 15;4(2):e22. doi: 10.1371/journal.pcbi.0040022 (PMC2242819; doi:10.1371/journal.pcbi.0040022)

Microarray Based Diagnosis Profits from Better Documentation of Gene Expression Signatures — *supplement* —

Dennis Kostka* and Rainer Spang†

* Max Planck Institute for Molecular Genetics
Ihnestrasse 63-73, 14195 Berlin, Germany

† Institute for Functional Genomics
Computational Diagnostics Group
Josef-Engert-Strasse 9, 93503 Regensburg, Germany

Contents

1 Confidence intervals for the consistency index **2**

2 Software **2**
2.1 Preprocessing 2
2.2 Building the classification rule 3
2.3 Documentation by value 3
2.4 Diagnosing an external patient 3

3 Supplement Figures **4**

1 Confidence intervals for the consistency index

In the context of the resampling experiment described in the paper, let there be n patients in the study, each predicted at least m times. For patients predicted more often, a random subsample of size m is taken. Let $\boldsymbol{p} \in \mathbb{R}^{nm}$ be the vector of predictions and $\boldsymbol{p}^\star$ the reference predictions. Then consistency is defined as

$$\bar{c} = \frac{1}{nm} \sum_{i}^{nm} \underbrace{\delta(\boldsymbol{p}_i - \boldsymbol{p}_i^\star)}_{\boldsymbol{c}_i}.$$

If the entries $\boldsymbol{c} = (\boldsymbol{c}_1, \ldots, \boldsymbol{c}_{nm})$ arise from *iid* Bernoulli variables with success-parameter p, then $nm \cdot \bar{c}$ is distributed binomially with parameters p and $N = nm$. Estimates for $\bar{c}$ and confidence intervals follow directely from the binomial distribution.

Confidence intervals for the consistency gain

The consistency gain or documentation effect is defined by $\bar{c}_\star := \bar{c}_{\text{val.}} - \bar{c}_{\text{ref.}}$. Assuming the same model as in the last paragraph, $nm \cdot \bar{c}_\star$ is the difference of two binomialy distributed random variables with parameters $nm, \bar{c}_{\text{val.}}$ and $\bar{c}_{\text{ref.}}$, respectively. Performing the convolution numerically yields confidence intervals.

2 Software

In this section we give a description of the software we provide as well as instructions how to use it. It is written in the `R` programming language [?] and makes use of packages contributed to the Bioconductor project [?], most notably `vsn` and `affy`, as well as of the `pamr`[?] package obtainable via `CRAN` [?]. The software we provide can be downloaded from `http://www.compdiag.molgen.mpg.de`.
To lead through the steps of building a signature documented by value, we assume a collection of `.cel`-files is located in a directory `"mydir"` awaiting analysis.

2.1 Preprocessing

Data are preprocessed using `rma` as well as `vsn` preprocessing. The variables `par.vsn` and `par.rma` hold the parameter sets determined in the theory section. We proceed in three steps:

i) Read in the data

```
> setwd("mydir")
> abo = ReadAffy()
```

ii) Preprocessing using `rma`

```
> exs.rma = wrap.pag(abo, method = "rma")
> par.rma = preproc(description(exs.rma))$pag
```

iii) Preprocessing using `vsn`

```
> exs.vsn = wrap.pag(abo, method = "vsn")
> par.vsn = preproc(description(exs.vsn))$pag
```

2.2 Building the classification rule

A classification rule is derived using `pamr`. The function `pamr.fil` is a simple wrapper, performing model selection based on crossvalidation-error. The vector `my.labels` holds class information for the study data.

```
> lab = my.labels
> sig.vsn = pamr.fil(exs.vsn, lab, fil = FALSE)
> sig.rma = pamr.fil(exs.rma, lab, fil = FALSE)
```

2.3 Documentation by value

We document the classification rules by value: The signature, together with the data-dependent scale information, is stored in a binary format for later application to external samples:

```
> sig.byval.rma = list(sig = sig.rma, params = par.rma)
> sig.byval.vsn = list(sig = sig.vsn, params = par.vsn)
> save(sig.byval.rma, file = "sig_byval_rma.rdat")
> save(sig.byval.vsn, file = "sig_byval_vsn.rdat")
```

When publishing the signature, the file `"sig_byval_rma.rdat"` or `"sig_byval_vsn.rdat"` can be made available on supplemental web-pages.

2.4 Diagnosing an external patient

We diagnose an external patient (`external_patient.CEL.gz`). Again, we proceed in three steps:

i) Read in the data

```
> abo.extrnl = ReadAffy("external_patient.CEL.gz")
```

ii) Add-on preprocess the data, transforming it to a study-consistent scale. The function `wrap.pag.add` utilizes the results from the theory section. In order to do that, data-dependent information stored with the signature has to be retrieved.

```
> load("sig_byval_rma.rdat")
> load("sig_byval_vsn.rdat")
> exs.extrnl.rma = wrap.pag.add(abo.extrnl, sig.byval.rma$params,
+     method = "rma")
> exs.extrnl.vsn = wrap.pag.add(abo.extrnl, sig.byval.vsn$params,
+     method = "vsn")
```

iii) Predict the class labels of the external patient, using the classifier derived beforehand:

```
> diag.rma = sig.byval.rma$sig(exprs(exs.extrnl.rma))
> diag.vsn = sig.byval.rma$sig(exprs(exs.extrnl.vsn))
```

3 Supplement Figures

In the following we show figures displaying consistency and kappa indices for each data set. We show all possible combinations of classifiers (pam,svm), size (20,30,40), preprocessing protocol (`rma`,`vsn`) and strategy (by reference, by value). The dashed lines always depict the mean values.

Figure S1: Beer

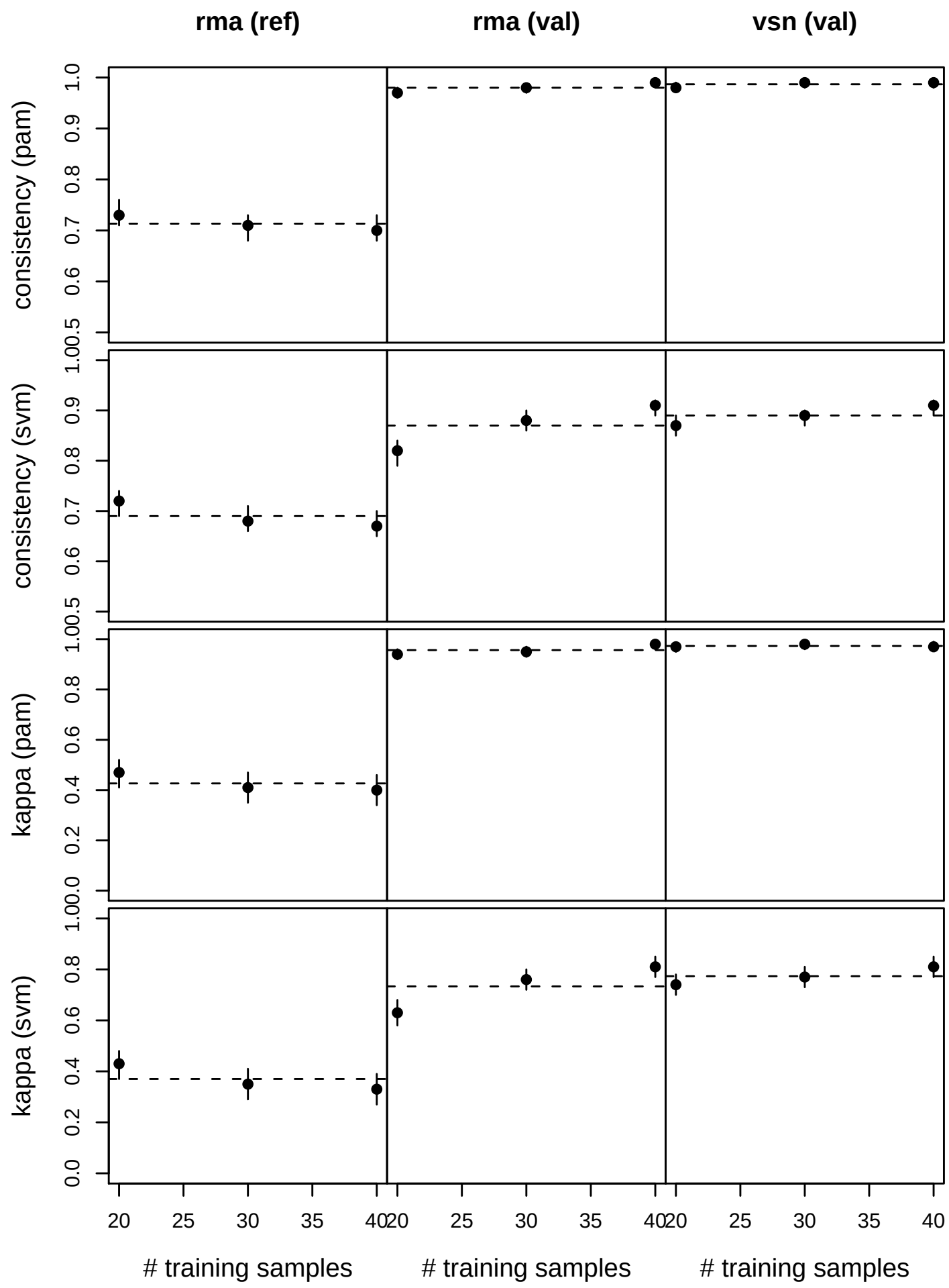

Figure S2: Bhattaharjee

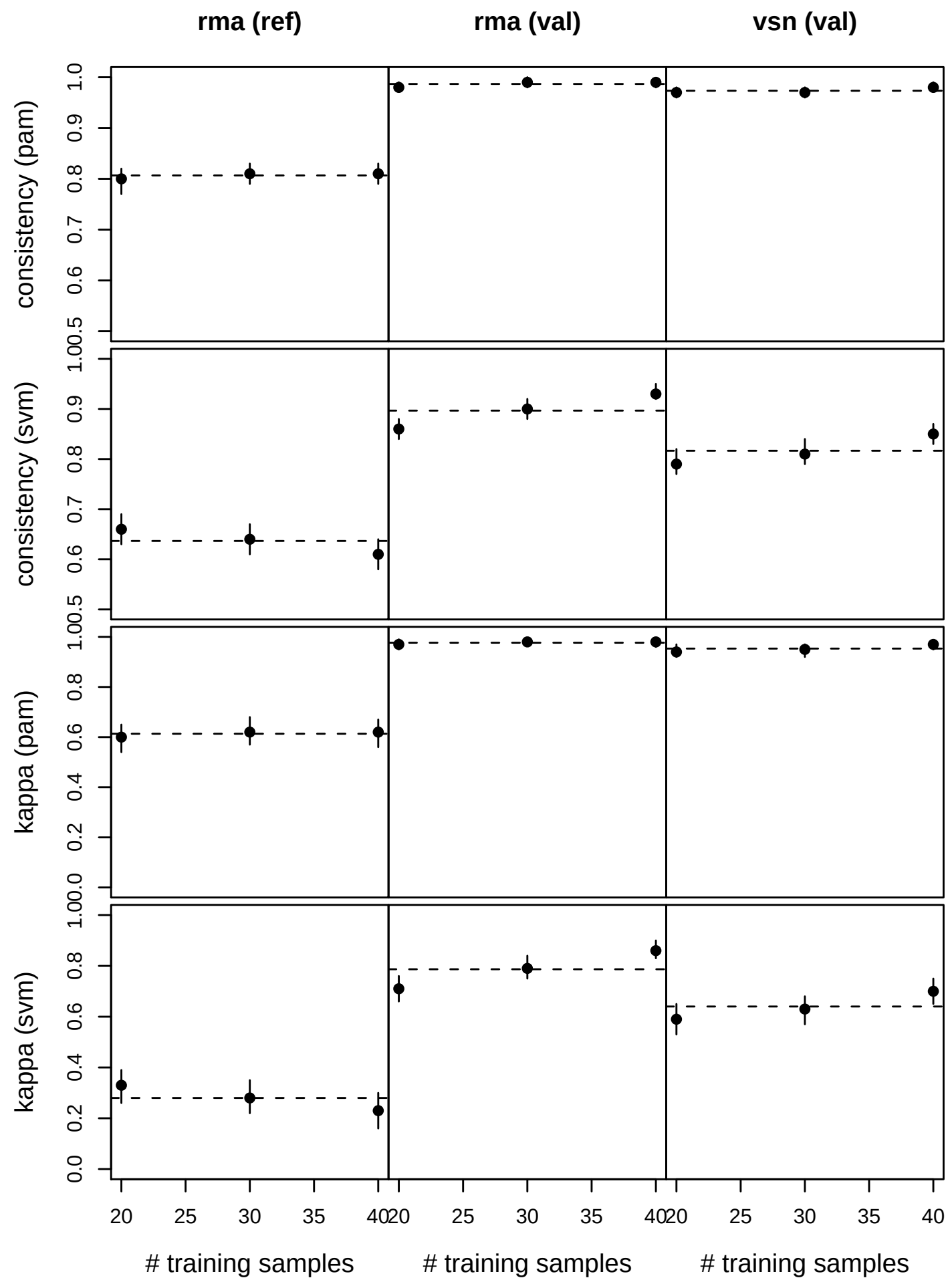

Figure S3: Bild

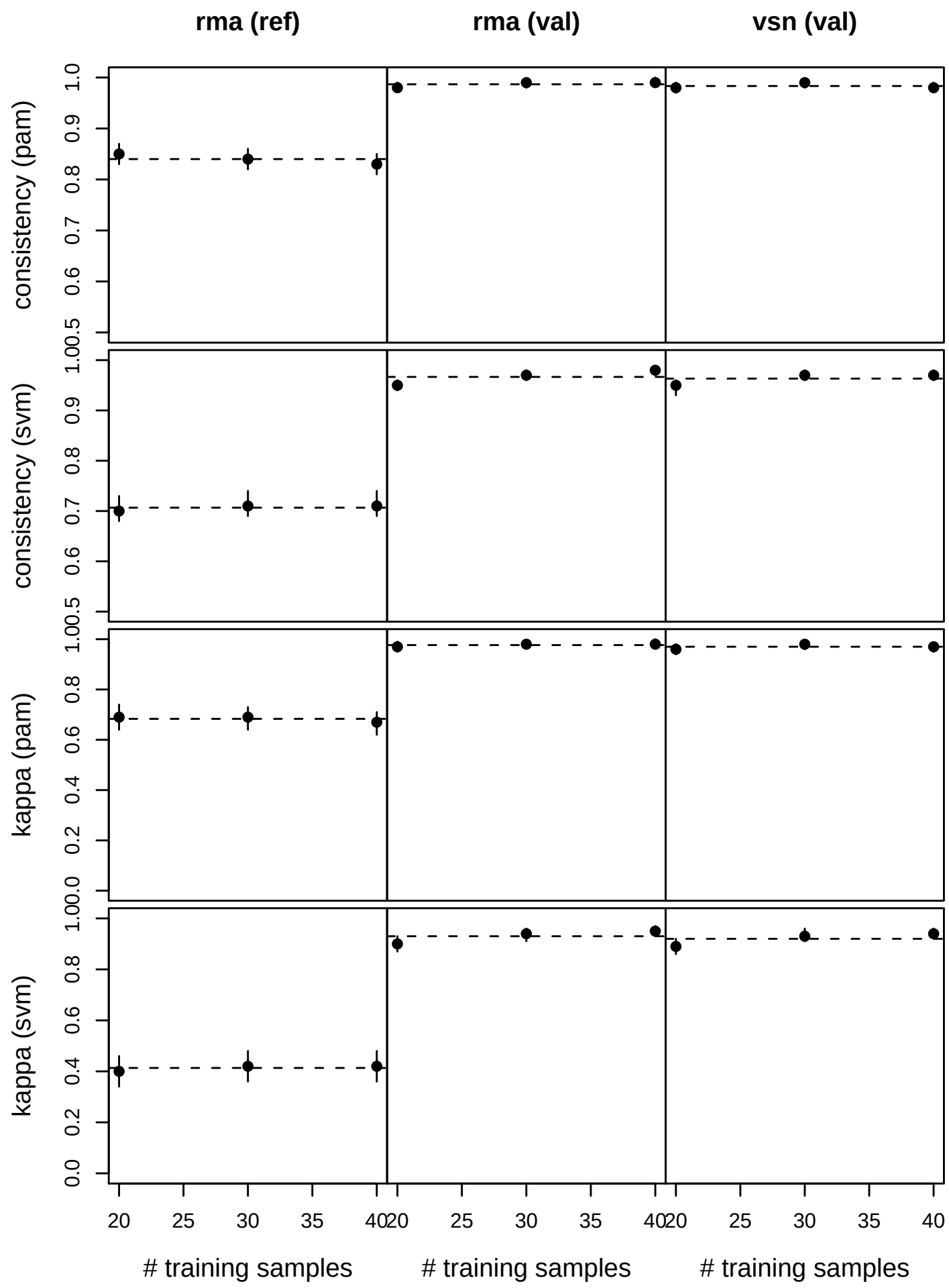

Figure S4: Huang

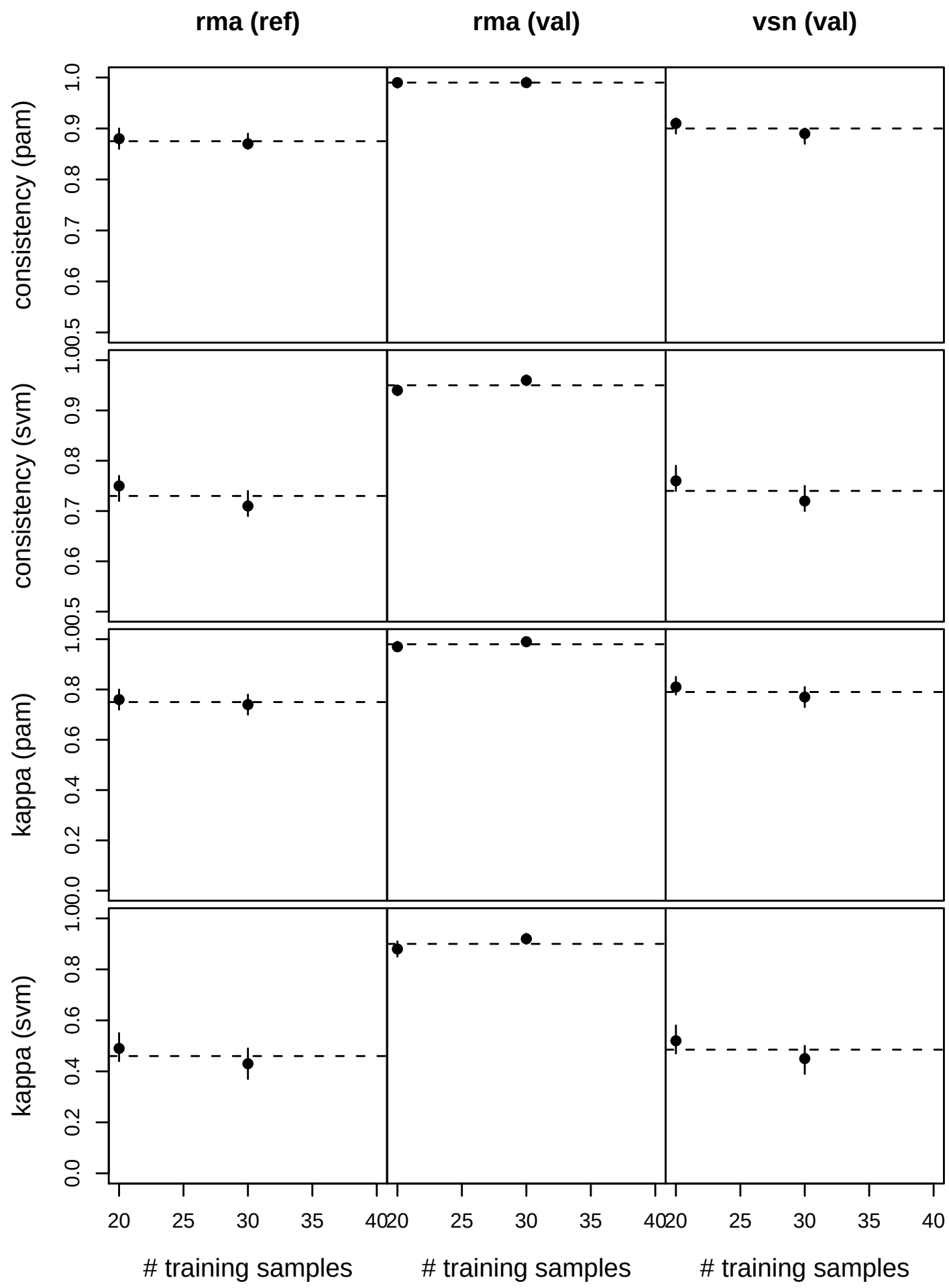

Figure S5: Pomeroy

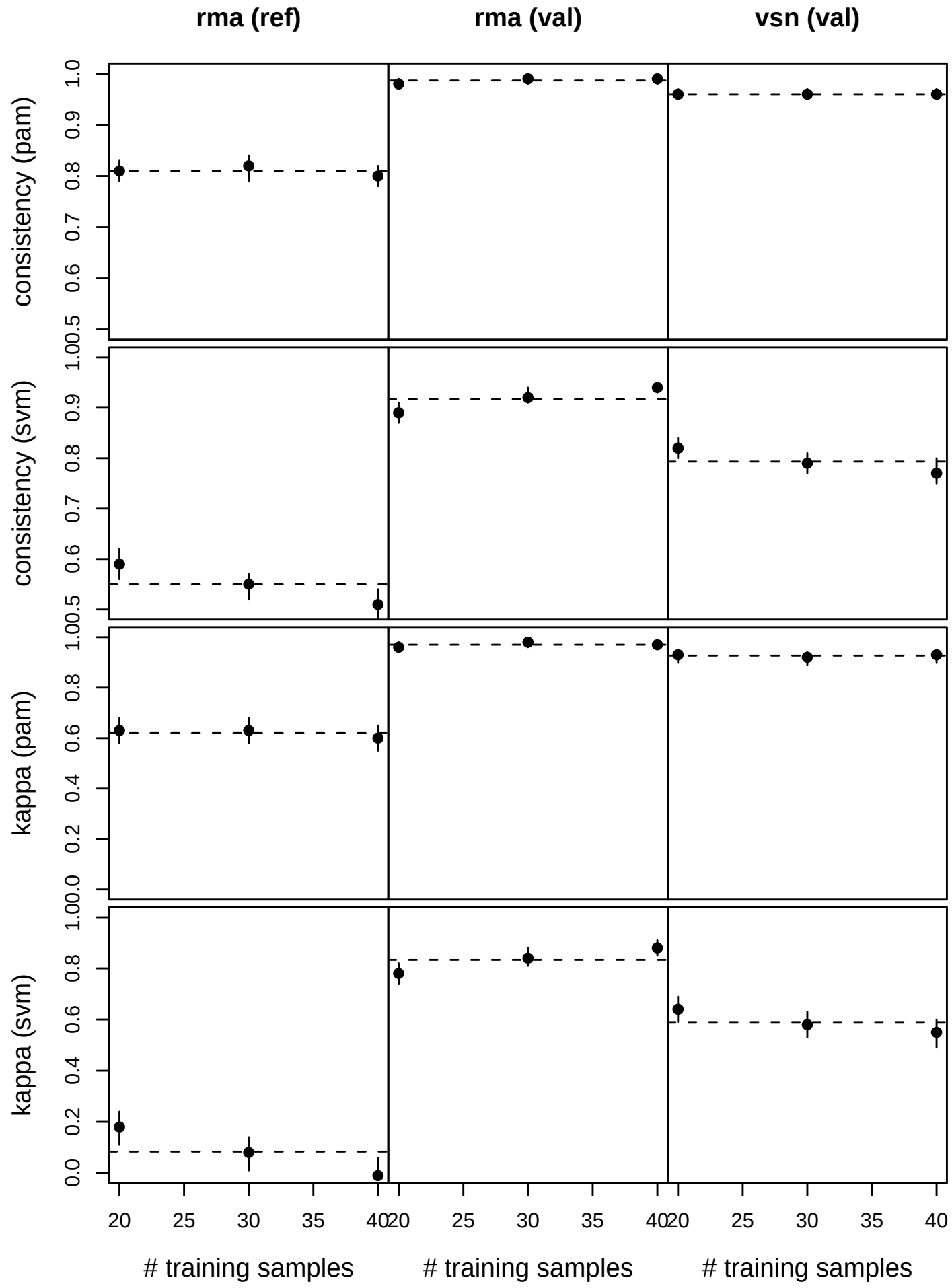

Figure S6: Ross

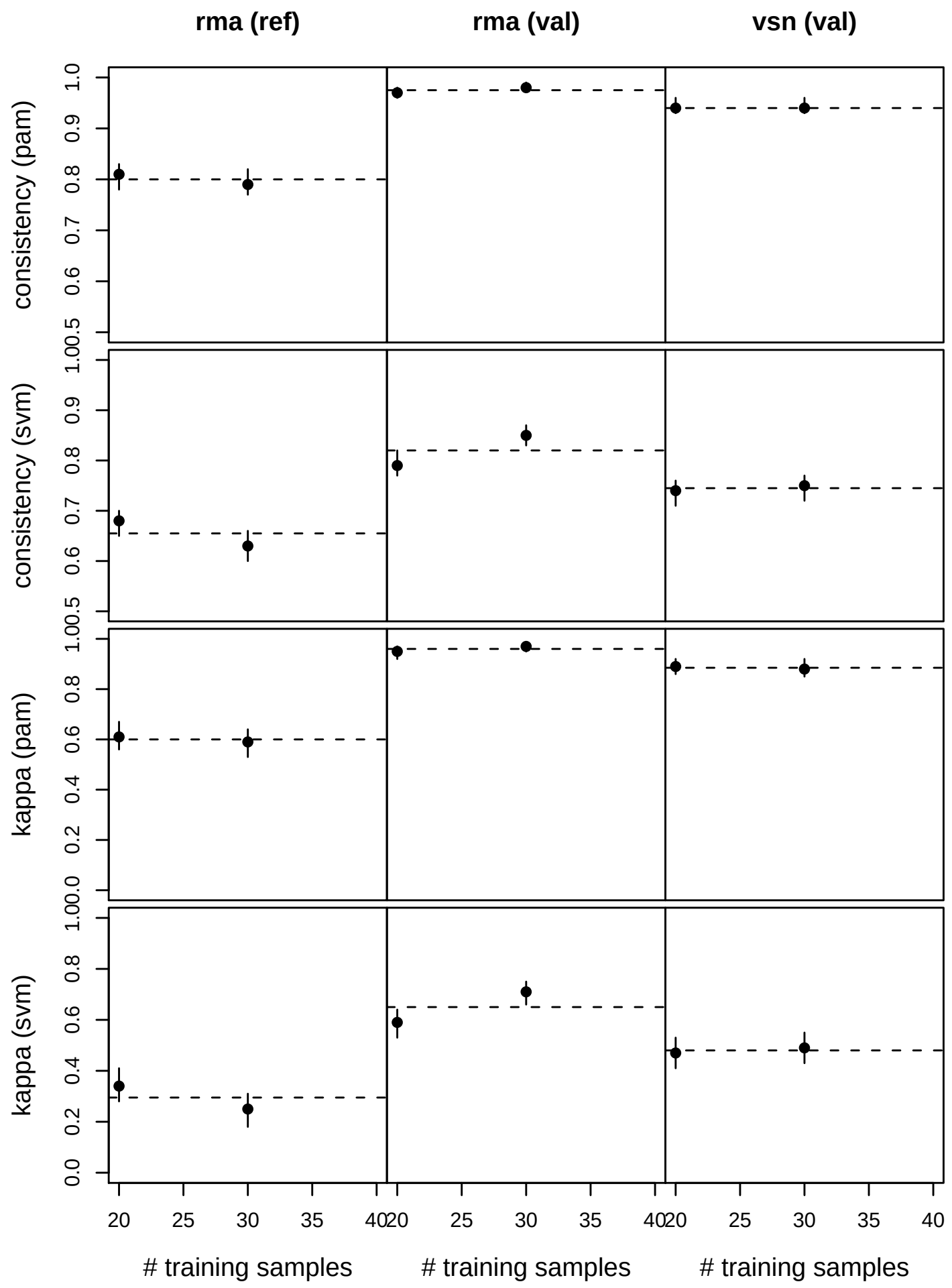

Figure S7: Shipp

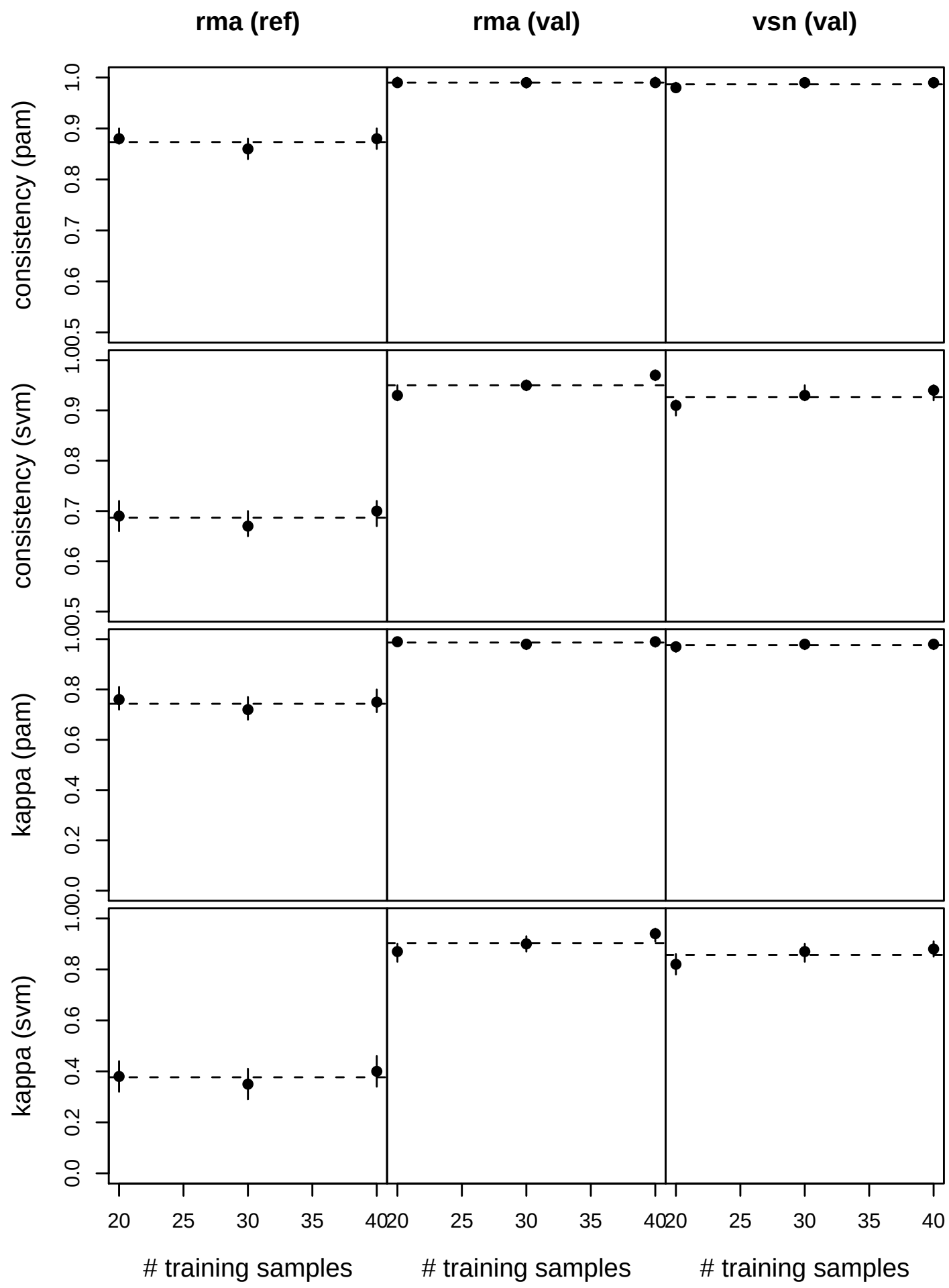

Figure S8: Willenbrock

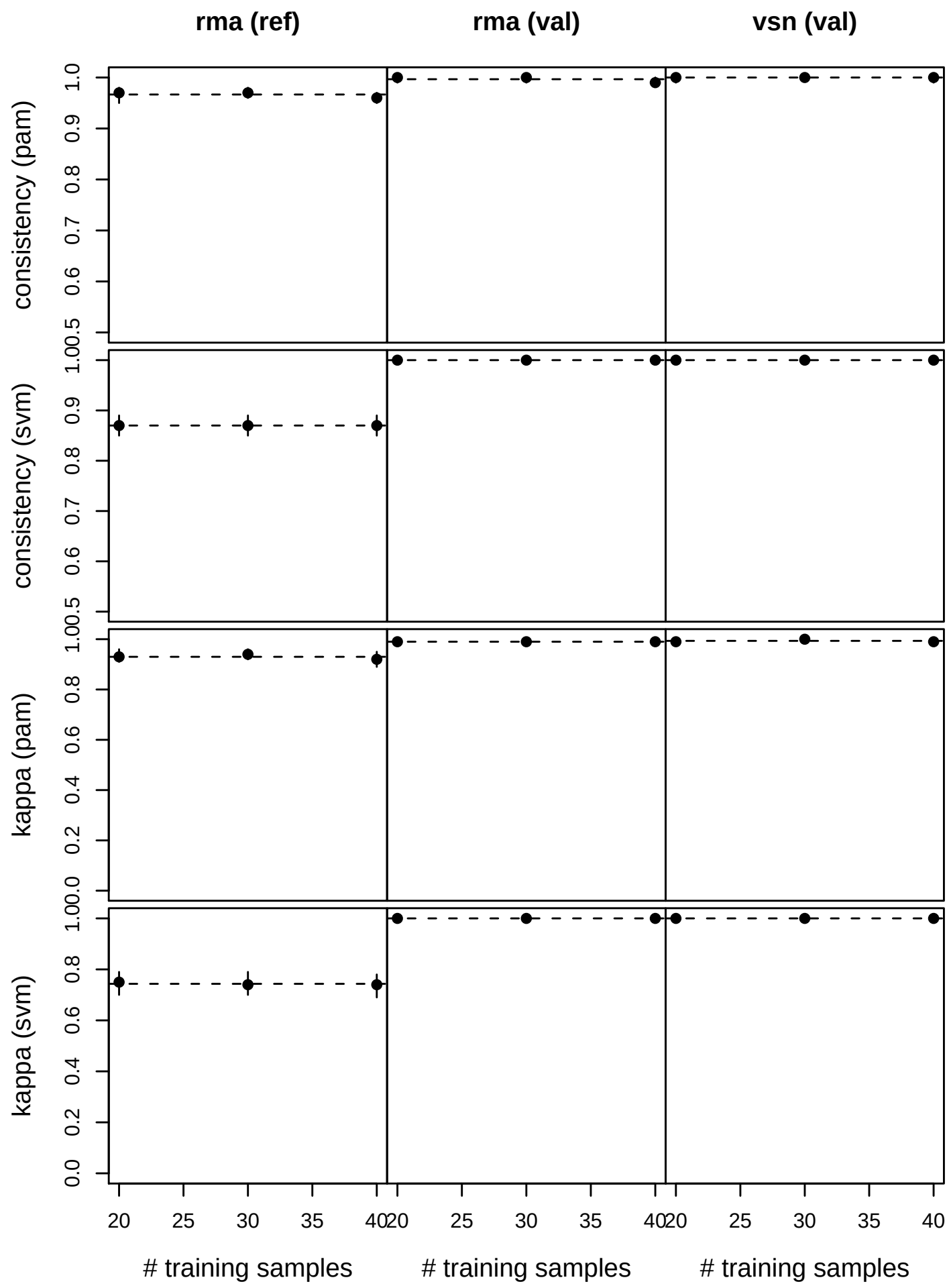

Supplement: Supplementary file 1 [file pcbi.0040022.sd001.pdf]
