# Supplementary material for: Microarray Based Diagnosis Profits from Better Documentation of Gene Expression Signatures
Source: PLoS Comput Biol. 2008 Feb 15;4(2):e22. doi: 10.1371/journal.pcbi.0040022 (PMC2242819; doi:10.1371/journal.pcbi.0040022)
Supplement: Supplementary file 4 [file pcbi.0040022.sg003.pdf]

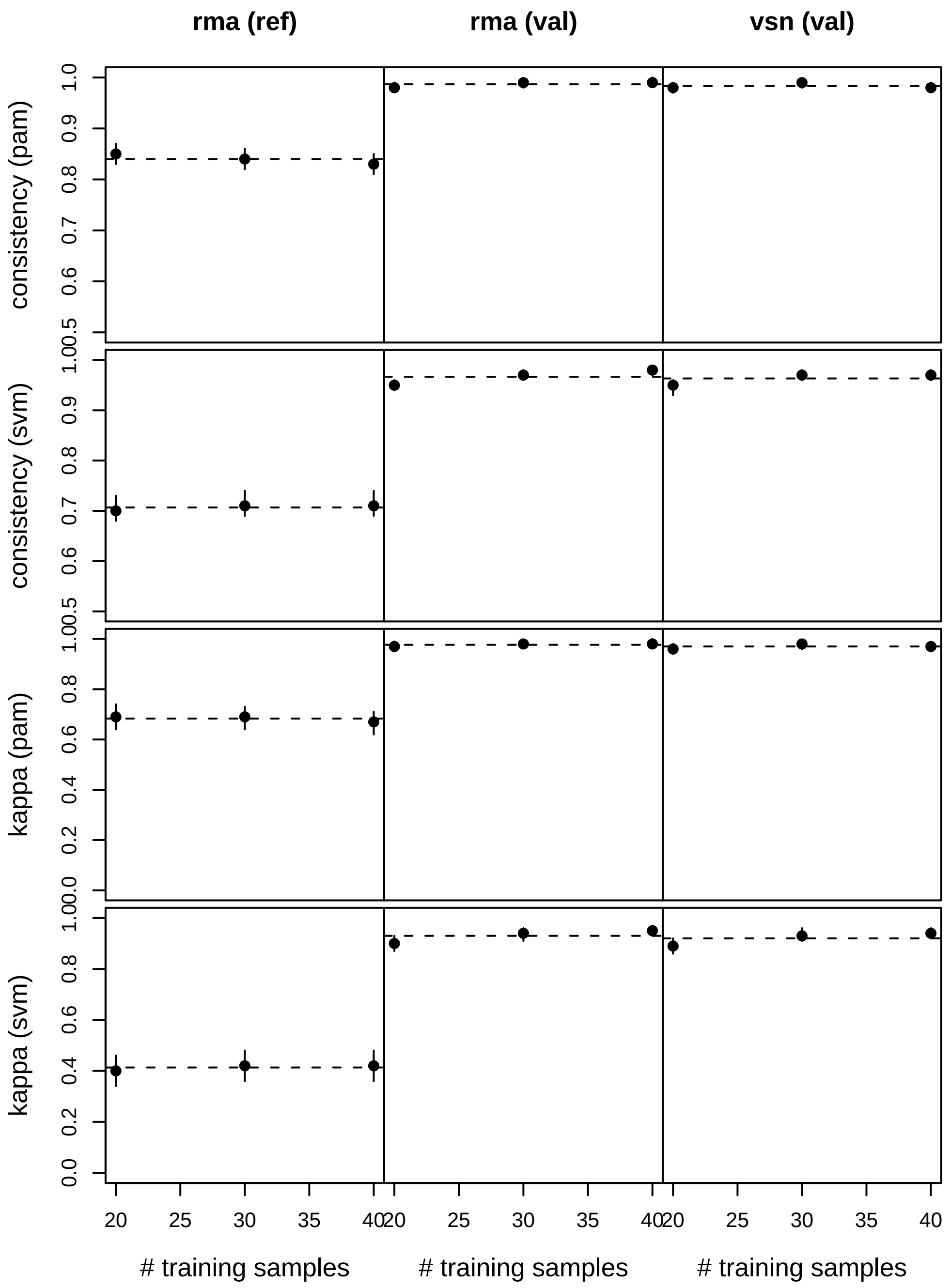
rma (ref)
rma (val)
vsn (val)
consistency (pam)
consistency (svm)
kappa (pam)
kappa (svm)
1.0
0.9
0.8
0.7
0.6
0.5
1.00
0.8
0.6
0.4
0.2
0.0
20
25
30
35
40
training samples
training samples
training samples
